# Constituents of *Coreopsis lanceolata* Flower and Their Dipeptidyl Peptidase IV Inhibitory Effects

**DOI:** 10.3390/molecules25194370

**Published:** 2020-09-23

**Authors:** Bo-Ram Kim, Sunil Babu Paudel, Joo-Won Nam, Chang Hyun Jin, Ik-Soo Lee, Ah-Reum Han

**Affiliations:** 1 Advanced Radiation Technology Institute, Korea Atomic Energy Research Institute, Jeongeup-si, Jeollabuk-do 56212, Korea; boram1606@kaeri.re.kr (B.-R.K.); chjin@kaeri.re.kr (C.H.J.); 2 College of Pharmacy, Chonnam National University, Gwangju 61186, Korea; 3 College of Pharmacy, Yeungnam University, Gyeongsan-si, Gyeongsangbukdo 38541, Korea; phrsunil@gmail.com (S.B.P.); jwnam@yu.ac.kr (J.-W.N.)

**Keywords:** *Coreopsis lanceolata*, polyacetylene glycoside, flavonoid, dipeptidyl peptidase IV, type 2 diabetes mellitus.

## Abstract

A new polyacetylene glycoside, (5*R*)-6*E*-tetradecene-8,10,12-triyne-1-ol-5-*O*-*β*-glucoside (**1**), was isolated from the flower of *Coreopsis lanceolata* (Compositae), together with two known compounds, bidenoside C (**10**) and (3*S*,4*S*)-5*E*-trideca-1,5-dien-7,9,11-triyne-3,4-diol-4-*O*-*β*-glucopyranoside (**11**), which were found in *Coreopsis* species for the first time. The other known compounds, lanceoletin (**2**), 3,2′-dihydroxy-4-3′-dimethoxychalcone-4′-glucoside (**3**), 4-methoxylanceoletin (**4**), lanceolin (**5**), leptosidin (**6**), (2*R*)-8-methoxybutin (**7**), luteolin (**8**) and quercetin (**9**), were isolated in this study and reported previously from this plant. The structure of **1** was elucidated by analyzing one-dimensional and two-dimensional nuclear magnetic resonance and high resolution-electrospray ionization-mass spectrometry data. All compounds were tested for their dipeptidyl peptidase IV (DPP-IV) inhibitory activity and compounds **2**-**4**, **6** and **7** inhibited DPP-IV activity in a concentration-dependent manner, with IC_50_ values from 9.6 to 64.9 μM. These results suggest that *C. lanceolata* flower and its active constituents show potential as therapeutic agents for diseases associated with type 2 diabetes mellitus.

## 1. Introduction

*Coreopsis* is a perennial plant belonging to the Compositae family [1]. It was planted for decoration on the roadsides in North America, Asia and Oceania regions [2,3,4]. Typically, *C. lanceolata*, *C. tinctoria* and *C. drummondii* are generally *Coreopsis* plants distributed all around Korea [5]. Among them, *C. lanceolate* are ornamental plants commonly found in Korea in spring and summer. The shape of the yellow petals are split and jagged, and have a diameter 4-6 cm, which is larger than other species [5,6]. In the previous phytochemical studies on *C. lanceolata*, phenolics, flavonoids (aurones, chalcones, flavaons, flanavol) and acetylene compounds have been isolated from this plant [5,6,7,8,9,10,11,12]. The *C. lanceolata* flower has been reported to have diverse biological activities, such as anti-cancer [5], anti-inflammatory [6], antioxidant [6,8,10], anti-allerginc [9,10], antileukemic [11] and nematicidal [12] effects. Because of the good efficacy of *C. lanceolata*, it has been traditionally used as an ingredient in herbal medicine, such as folk remedies to control fever in China and East Asia [8]. This plant has been studied for a few chemical constituents and its pharmacological activity, but a literature review revealed that there is no anti-diabetes.

Type 2 diabetes mellitus is a chronic metabolic disorder, characterized by hyperglycemia caused by the dysregulation of blood glucose homeostasis, increased liver glucose production, insulin secretion, insulin resistance and β-cell dysfunction [13]. According to the eighth edition of the International Diabetes Federation (IDF) Diabetes Atlas, there were 425 million people with diabetes worldwide in 2017, and most of them have type 2 diabetes mellitus. This number of patients is increasing every year, and is expected to increase to about 700 million by 2045 [14]. The five factors that affect diabetes treatment are dipeptidyl peptidase- IV (DPP-IV), α-glucosidase inhibitors, aldose reductase (ALR) inhibitors, phatase 1B (PTP1B) inhibitors and peroxisome proliferator-activated receptor-γ (PPAR-γ) [15]. Recently, incretin-based therapies for the treatment of type 2 diabetes mellitus have begun to appear, one of which is DPP-IV inhibitors [16]. The incretin system includes glucagon-like peptide-1 (GLP-1) and glucose-dependent insulinotropic polypeptide (GIP), which in response to high blood sugar levels induce the release of insulin from the pancreatic β-cell [13,16]. DPP- IV is involved in the quick degradation and inactivation of GLP-1 and GIP in the incretin system [17]. As a result, DPP-IV inhibition enhances the glucose-producing effect of GLP-1, thereby improving glucose tolerance in diabetic patients [18]. As DPP-IV inhibitors, synthetic chemical compounds or natural product-derived compounds recently represent promising drug candidates [16,19,20,21,22].

**Figure 1:**
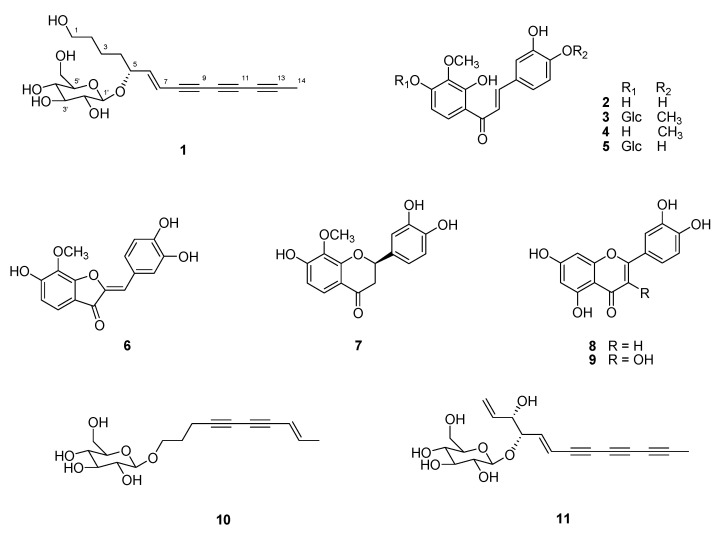
Chemical structures of compounds isolated from the ethyl acetate-soluble fraction of *Coreopsis lanceolata*.

During the screening procedure to find new candidates from natural sources for the treatment of type 2 diabetes mellitus, the ethyl acetate fraction of the *C. lanceolata* flower showed potent inhibitory activity in a DPP-IV inhibitor screening assay, with 100% inhibition at a concentration of 100 μg/mL. Therefore, this active fraction was subjected to detailed phytochemical investigation, and 11 compounds were isolated, including a new compound **1** (Figure 1). In this study, we describe the isolation and structural elucidation of **1** and the biological results of compounds **1**–**11**.

## 2. Results and Discussion

### 2.1. Structure Elucidation of Compound 1 

**Figure 2:**
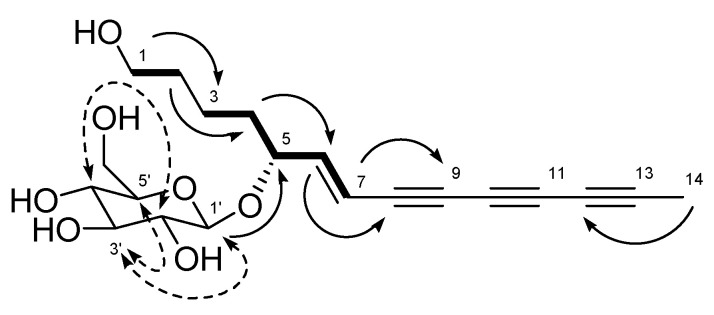
Key ^1^H-^1^H COSY (**–**), ^1^H-^1^H NOESY (----), and ^1^H-^13^C HMBC (→) correlations of **1**.

Compound **1** was obtained as a brown solid, with a molecular ion peak at m/z 401.1575 [M+Na]^+^ in the high resolution electrospray ionization mass spectrum, corresponding to an elemental formula of C_20_H_26_O_7_Na. Its specific UV spectrum showed maximum absorptions at 254, 260, 274, 290, 310 and 330 nm, predicted to be ene-triyne chromophore [23,24]. The ^1^H NMR and ^13^C NMR spectra of **1** (Table 1) exhibited signals for a trans-olefin group at δ_H_ 6.39 (1H, dd, *J* = 16.0, 6.0 Hz, H-6)/δ_C_ 151.4 (C-6) and δ_H_ 6.06 (1H, dd, *J* = 16.0, 1.5 Hz, H-7)/δ_C_ 108.6 (C-7), three methylene groups at δ_H_ 1.45 (2H, dd, *J* = 13.0, 6.8 Hz, H-4)/δ_C_ 35.1 (C-4), δ_H_ 1.35 (2H, dd, *J* = 14.0, 6.3 Hz, H-2)/δ_C_ 32.8 (C-2), and δ_H_ 1.28 (2H, dd, *J* = 14.0, 6.8 Hz, H-3)/δ_C_ 21.7 (C-3), an oxygenated methylene group at δ_H_ 3.33 (2H, dd, *J* = 12.3, 6.3 Hz, H-1)/δ_C_ 61.1 (C-1), and an oxygenated methine group at δ_H_ 4.26 (1H, ddd, *J* = 13.0, 6.0, 1.5 Hz, H-5)/δ_C_ 76.5 (C-5), representing a 6-heptene-1,5-diol group, which was supported by the ^1^H-^1^H COSY correlations of H-1/H-2, H-4/H-3, H-5 and H-6/H-5, H-7, and the ^1^H-^13^C HMBC correlations of H-1/C-3, H-2/C-4 and H-4/C-6 (Figure 2). Additional signals for a methyl group at δ_H_ 2.02 (3H, s, H-14)/δc 4.6 (C-14) and six acetylene carbons at δc 81.3 (C-13), 75.4 (C-8), 74.0 (C-9), 64.6 (C-12), 67.4 (C-11) and 59.0 (C-10) indicated the presence of a 1,3,5-heptatriyne group, which was attached at the trans-olefin group by the ^1^H-^13^C HMBC NMR correlations of H-7/C-9 and H-14/C-12. The ^1^H NMR and ^13^C NMR spectra of **1** also displayed signals for sugar moiety at δc 77.4 (C-5′), 77.3 (C-3′), 74.0 (C-2′), 70.6 (C-4′) and 61.7 (C-6′), including the anomeric signal at δ_H_ 4.00 (1H, d, *J* = 8.0 Hz, H-1′)/δc 101.3 (C-1′), and the sugar moiety was determined as *β*-glucopyranoside by the ^1^H-^1^H NOESY correlations of H-1′/H-3′ and H-5′, and H-2′/H-4′ [25,26]. In the ^1^H-^13^C HMBC NMR spectrum, the hemiacetal proton H-1′ showed a key correlation with C-5, suggesting that the *β*-glucopyranoside was positioned at C-5 in the polycetylene aglycone. An absolute configuration of **1** at C-5 was assigned by comparing its experimental circular dichroism (CD) data with the literature data [27]. In the literature, polyacetylene glycosides established their absolute configurations by comparison of the calculated and experimental ECD spectra. According to the Snatzke′s helicity rule, the negative Cotton effect at 300–320 nm permitted the configuration of the glucoside-attached carbon to *R*, while the positive Cotton effect at 300–320 nm permitted the configuration of the glucoside-attached carbon to *S*. Based on this observation (Figures S8 and S9), the absolute configuration of **1** was determined as (5*R*). Therefore, the structure of **1** was elucidated as (5*R*)-6*E*-tetradecene-8,10,12-triyne-1-ol-5-*O*-*β*-glucoside (**1**), which is a new polyacetylene glycoside.

The 10 known compounds were identified as lanceoletin (**2**) [11], 3,2′-dihydroxy-4-3′-dimethoxychalcone-4′-glucoside (**3**) [8], 4-methoxylanceoletin (**4**) [11], lanceolin (**5**) [10], leptosidin (**6**) [11], (2*R*)-8-methoxybutin (**7**) [11], luteolin (**8**) [28], quercetin (**9**) [29], bidenoside C (**10**) [30] and (3*S*,4*S*)-5*E*-trideca-1,5-dien-7,9,11-triyne-3,4-diol-4-*O*-*β*-glucopyranoside (**11**) [31] by comparing their spectroscopic data with published data. Flavanone **7** showed the negative Cotton effect at 295–310 nm and the positive Cotton effect at 280–290 nm in its CD spectrum, indicating a 2*R* configuration [32] (Figure S10). The absolute configuration of **11** at C-3 and C-4 was assigned by comparing its experimental CD data with the data in the literature [27], as described above for the absolute configuration determination of **1**. Compound **11** was a diastereomer and the threo stereochemistry between H-3 and H-4 was deduced by their large coupling constant (*J* = 5.5 Hz, in methanol-*d*_4_). The positive Cotton effect at 300–320 nm in the CD spectrum of **11** was attributed to a (3*S*,4*S*) configuration for **11** (Figure S11). Although the known compounds (**2**-**9**) have been isolated from the *Coreopsis* species, the isolation of compounds **10** and **11** from the *Coreopsis* species has not been reported yet.

### 2.2. Biological Activity

**Figure 3:**
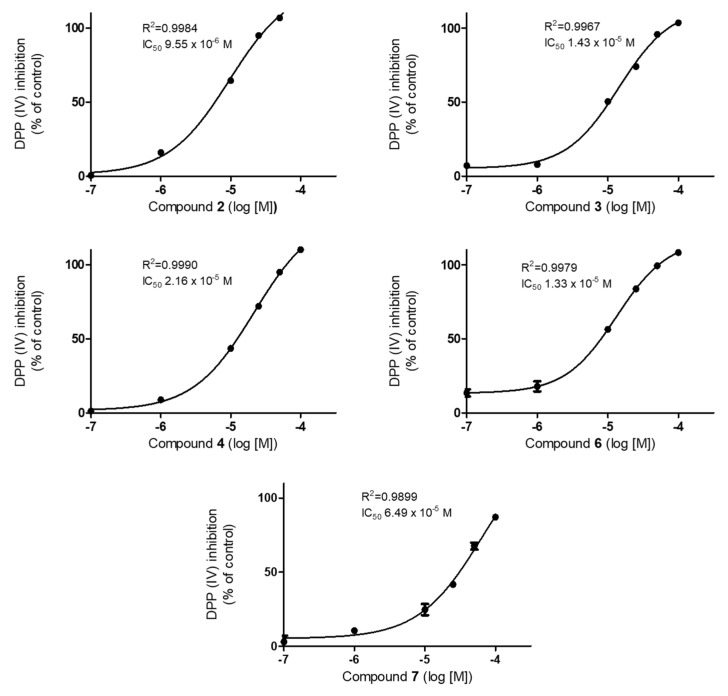
Effects of compounds **2**–**4, 6** and **7** on DPP-IV activity. Values are presented as the mean ± SD of three independent experiments.

The methanol extract and solvent fractions of *C. lanceolata* confirmed their anti-diabetic effect using an in vitro DPP-IV inhibitor screening assay. The methanol extract inhibited DPP-IV activity with 87.2% inhibition at 100 μg/mL. Thus, it was partitioned with hexanes, ethyl acetate and *n*-butanol, successively, and these solvent fractions were screened for their DPP-IV inhibition at 100 μg/mL concentrations. Since the ethyl acetate fraction inhibited DPP-IV activity with 100% inhibition, we conducted a detailed phytochemical investigation, leading to the isolation of 11 compounds. All isolates were evaluated for their DPP-IV inhibitory effects, and compounds **2**–**4**, **6** and **7** inhibited DPP-IV activity in a concentration-dependent manner, with IC_50_ values of 9.6, 14.3, 21.6, 13.3 and 64.9 μM, respectively (Figure 3). The positive control, sitagliptin, exhibited an IC_50_ of 0.071 μM. 

Compounds **2**–**5** have the chalcone skeleton, which is an α-β-unsaturated ketone with two phenyl rings. There have been several reviews on the biological and pharmacological activities of chancones, including anti-cancer, anti-malarial, anti-microbial, anti-inflammatory, anti-protozoal and anti-HIV activities [33], as well as its role in diabetes control [15,34,35]. In a comprehensive study into modulating the therapeutic targets PPAR-γ, DPP-IV, α-glucosidase, PTP1B, ALR and insulin secretion via anti-diabetic chalcones and their structure–activity relationships [15], in particular, it was reported that the chalcone structure of which the carbonyl group was replaced with an oxime function exhibited enhanced DPP-IV inhibitory activity. In addition, there have been reports that nitrochalcones and prenylated chalcones increase insulin secretion and glucose uptake, respectively [34]. Among chalcones isolated in this study, **3**–**5** showed less inhibitory activity than **2**, indicating that chalcones substituted with additional methoxy group and/or sugar are less effective. The most active compound, **2,** has been reported as having various biological, activities such as antileukemic [11], antioxidant [4] and anti-cancer [5], however its DPP-IV potential has not been studied previously. Compounds **6** and **7,** with the structures of aurone and flavanone, respectively, exhibited moderate DPP-IV inhibitory effects, while flavone (**8**), flavonol (**9**) and polyacetylenes (**1**, **10** and **11**) did not show their activities. The evaluation of the inhibitory activities of **1**–**11** against DPP-IV was reported for the first time in this study.

Extensive studies on the phytochemical-based DPP-IV inhibitors were undertaken in the literature [19,20,21], and in our previous studies [16,22]. Flavonoids and phenolic compounds were found to be the DPP-IV inhibitors with potent to mild activities, showing IC_50_ values in the range of 0.12 to 63.26 μM. Although naturally occurring compounds were relatively less active than the synthetic DPP-IV inhibitors diprotin A and sitagiptin, the natural products with structural diversity could be useful for discovering safer DPP-IV inhibitors. Therefore, these results suggest that compounds **2**–**4**, **6** and **7** have the potential for the development of naturally derived DPP-IV inhibitors for the treatment of type 2 diabetes mellitus and hyperglycemia, although a further study of their mechanisms of action is necessary, using in vitro and in vivo models.

## 3. Materials and Methods 

### 3.1. General Procedures

The optical rotations were measured by a JASCO DIP-1000 polarimeter (JASCO Co., Tokyo, Japan). The circular dichroism (CD) measurements were conducted using a JASCO J-810 CD-ORD spectropolarimeter (JASCO Co., Tokyo, Japan). The 1D and 2D NMR specta were obtained from a JNM-ECA 500 MHz NMR instrument (JEOL Ltd., Tokyo, Japan). Tetramethylsilane (TMS) was used as an internal standard. High-resolution electrospray ionization mass spectra (HRESIMS) were obtained from a Waters SYNAPT G2 mass spectrometer (Waters, Milford, CT, USA). Column chromatography (CC) was performed on columns containing silica gel (70–230 mesh, Merck, Darmstadt, Germany), RP-18 (YMC gel ODS-A, 12 nm, S-75 μm, YMC Co., Tokyo, Japan) and Sephadex LH-20 (GE Healthcare Bio-Sciences, Uppsala, Sweden). Thin-layer chromatographic (TLC) analysis was performed on Kieselgel 60 F_254_ (Merck, Darmstadt, Germany) and Kieselgel 60 RP-18-F_254S_ (Merck, Darmstadt, Germany). Analytical plates were visualized under UV light (254 and 365 nm) and sprayed with 10% (*v*/*v*) sulfuric acid in water, followed by heating at 180 °C for 2 min. A YMC-Pack Pro C_18_ column (5 μm, 250 mm × 20 mm i.d., YMC Co. Tokyo, Japan) was utilized for preparative high performance liquid chromatography (HPLC), operated on a Gilson Preparative HPLC system (Gilson Inc., Middleton, WI, USA). Medium pressure liquid chromatography (MPLC) was performed on a CombiFlash Rf200 system (Teledyne ISCO, Lincoln, NE, USA) with Redi*Sep* Rf Normal Phase Silica columns.

### 3.2. Plant Material

*Coreopsis lanceolata* flowers were collected at the road side around Korea Atomic Energy Research Institute, Jeonguep-si, Jeollabuk-do 56212, Korea, in June 2017. These flowers were handpicked and randomly collected at the stage of fully open flowering, then were freeze-dried and stored at −20 °C in polyethylene plastic bags until further analysis. This plant was identified by Ah-Reum Han, one of the co-authors of this study. The voucher specimens (No. Con013) were deposited at the Radiation Breeding Research Center, Advanced Radiation Technology Institute, Korea Atomic Energy Research Institute.

### 3.3. Extraction and Isolation

Freeze-dried flowers (500 g) of *C. lanceolata* were extracted with MeOH (3 × 5 L) overnight at room temperature. The solvent was evaporated in vacuo to afford an MeOH extract (201.5 g) which was then suspended in distilled water (1 L) and partitioned with hexanes (3 × 1 L), ethyl acetate (3 × 1 L) and *n*-butanol (3 × 1 L) sequentially. The EtOAc-soluble fraction (25.2 g) was then subjected to reverse-phase-C18 column chromatography (RP-C18 CC) using a gradient solvent system of MeOH-H_2_O (1:1 to 1:0, *v*/*v*) to afford 9 fractions (F01–F09). Fraction F03 (1.7 g) was subjected to RP-C18 CC with a solvent system of MeOH-H_2_O (1:1 to 1:0, *v*/*v*), affording 13 subfractions (F0301-F0313). Subfraction F0306 (546 mg) was subjected to silica gel CC with a solvent system of CH_2_Cl_2_-MeOH (9:1 to 0:1, *v*/*v*) to produce 14 subfractions (F030601-F030614) together with purified compound **7** (2.7 mg). Subfraction F030614 was chromatographed on RP-C18 CC with a gradient solvent system of CH_3_CN-H_2_O (4:6 to 1:0, *v*/*v*) to obtain **3** (20 mg). Subfraction F0311 (44 mg) was purified by RP-C18 CC with a gradient solvent system of CH_3_CN-H_2_O (4:6 to 1:0, *v*/*v*) to yield **6** (1.0 mg). Fraction F07 (623 mg) was subjected to RP-C18 CC with a solvent system of CH_3_CN -H_2_O (4:6 to 1:0, *v*/*v*), affording 18 subfractions (F0701-F0718). Subfraction F0711 (20 mg) was chromatographed on a Sephadex LH-20 column using 100% MeOH to give **8** (3.0 mg) and **9** (2.5 mg). Subfraction F0705 (77 mg) was subjected to a Sephadex LH-20 column using 100% MeOH, affording six subfractions (F0701-F0706). Subfraction F070505 (25 mg) was chromatographed on silica gel CC with a solvent system of CH_2_Cl_2_-MeOH-H_2_O (9:1:0.1 to 0:1:0, *v*/*v*) to obtain **5** (2.5 mg). Subfraction F0708 (55 mg) was subjected to a Sephadex LH-20 column using 100% MeOH, affording six subfractions (F0701-F0706). Subfraction F0708 (55 mg) was subjected to a Sephadex LH-20 column using 100% MeOH to obtain **10** (2.0 mg). F09 (900 mg) was separated by MPLC (CHCl_3_-MeOH, 9:1 to 0:1, 15 mL/min), affording 16 subfractions (F0901-F0916). Subfraction F0901 was purified by Sephadex LH-20 CC using 100% MeOH to yield **4** (6.0 mg). Subfraction F0904 (21 mg) was chromatographed on a Sephadex LH-20 column using 100% MeOH to give **2** (2.7 mg). Subfraction F0910 (22 mg) was purified by a Sephadex LH-20 column using 100% MeOH to give **11** (4.0 mg). Subfraction F0912 (28 mg) was chromatographed on a Sephadex LH-20 column using 100% MeOH to give **1** (2.0 mg).

(5*R*)-6*E*-tetradecene-8,10,12-triyne-1-ol-5-*O*-*β*-glucoside (**1**): Brown solid. [α]_D_^26^ –11.5° (*c* 0.13, MeOH). CD (MeOH) λ_max_ (Δε) 228 (+0.52), 243 (–0.83), 286 (–0.11), 307 (–0.26) nm. ^1^H (500 MHz) and ^13^C (125 MHz) data (CD_3_OD), see Table 1 and Figures S1–S6. HRESIMS (positive ions) m/z 401.1575 [M+Na]^+^ (calculated for C_20_H_26_O_7_Na, 401.1575) (Figure S7).

(2*R*)-methoxybutin (**7**): Yellow powder. [α]_D_^26^ –117.9° (*c* 0.09, MeOH). CD (MeOH) λ_max_ (Δε) 275 (–0.11), 289 (+0.19), 300 (−0.23) nm.

(3*S*,4*S*)-5*E*-trideca-1,5-dien-7,9,11-triyne-3,4-diol-4-*O*-*β*-glucopyranoside (**11**): Brown solid. [α]_D_^26^ +82.1° (*c* 0.09, MeOH). CD (MeOH) λ_max_ (Δε) 226 (−2.50), 253 (+1.33), 290 (+0.67), 309 (+0.78) nm.

### 3.4. DPP-IV Inhibitory Activity Assay

The measurement of DPP-IV activity was performed using the DPP-IV inhibitor screening assay kit (Cayman Chemical, Ann Arbor, Michigan, USA), based on the manufacturer’s protocols (Cayman Chemical). Briefly, a DDP assay buffer consisting of 20 mM Tris-HCL (pH 8.0), 100 nM NaCl and 1 mM EDTA was used for the assay solution. Each of the compounds were initially dissolved in DMSO at a concentration of 1 mM. Subsequently, the final concentration of compounds was diluted to 0.1, 1, 10, 25, 50 and 100 μM, respectively. The human recombinant DPP-IV enzyme and the substrate 5 mM H-Gly-Pro conjugated to aminomethylcoumarin (AMC) were put in the same buffer. Diluted assay buffer (30 μL) and diluted enzyme solution (10 μL) were added to a 96-well plate containing 10 μL of solvent (blank) or 10 μL of solvent-dissolved test compounds. The reaction by adding 50 μL of the diluted solution of the fluorogenic substrate was initiated and cleavage of the peptide bond by DPP released the free AMC group. The fluorescence was measured as an excitation wavelength of 350 nm and an emission wavelength of 450 nm using a plate reader (TECAN, Männedorf, Switzerland). The percentage inhibition was expressed as [(DPP-IV level of vehicle-treated control−DPP-IV level of test samples)/DPP-IV level of vehicle-treated control] × 100. Subsequently, the 50% inhibitory concentration (IC_50_) was determined using GraphPad Prism software (GaraphPad Software, La Jolla, CA, USA) via a dose–response analysis.

## 4. Conclusions

A new polyacetylene glycoside, namely (5*R*)-6*E*-tetradecene-8,10,12-triyne-1-ol-5-*O*-*β*-glucoside (**1**), was isolated from the *C. lanceolata* flower, along with 10 known compounds. Other polyacetylene glycosides, bidenoside C (**10**) and (3*S*,4*S*)-5*E*-trideca-1,5-dien-7,9,11-triyne-3,4-diol-4-*O*-*β*-glucopyranoside (**11**), were found in the *Coreopsis* species for the first time. In the screening for any DPP-IV inhibitory activity of the isolates **1–11**, compounds **2**–**4**, **6** and **7** inhibited DPP-IV enzyme activity in a dose-dependent manner. This study provides the first demonstration of the compounds responsible for the DPP-IV inhibitory activity of the *C. lanceolata* flower. Moreover, lanceoletin (**2**) exhibited the most DPP- IV inhibitory effect; thus our results suggest that **2** could be considered as a lead compound for the development of agents against DPP-IV activity.

## Figures and Tables

**Table 1:** ^1^H-NMR (500 MHz) and ^13^C-NMR (125 MHz) spectral data (DMSO-*d*_6_, δ in ppm) of **1** isolated from *Coreopsis lanceolata*.

Position	1		
	δ_H_	δ_C_	HMBC (carbon no.)
1	3.33 (2H, dd, *J* = 12.3, 6.3)	61.1	3
2	1.35 (2H, dd, *J* = 14.0, 6.3)	32.8	4
3	1.28 (2H, dd, *J* = 14.0, 6.8)	21.7	5
4	1.45 (2H, dd, *J* = 13.0, 6.8)	35.1	6
5	4.26 (1H, ddd, *J* = 13.0, 6.0, 1.5)	76.5	1′, 7
6	6.39 (1H, *J* = dd, *J* = 16.0, 6.0)	151.4	8
7	6.06 (1H, dd, *J* = 16.0, 1.5)	108.6	9, 10
8		75.5	
9		74.0	
10		59.0	
11		67.4	
12		64.6	
13		81.3	
14	2.02 (3H, s)	4.6	10, 11, 12
1′	4.00 (1H, d, *J* = 8.0)	101.3	5, 6
2′	2.92 (1H, dd, *J* = 8.0, 4.5)	74.0	
3′	3.07 (1H, dd, *J* = 8.7, 4.5)	77.3	
4′	2.99 (1H, m)	70.6	
5′	2.99 (1H, m)	77.4	
6′	3.38 (1H, m) 3.61 (1H, dd, *J* = 11.4, 6.0 Hz)	61.7	

## Supplementary Materials

The following are available online, Figure S1: ^1^H NMR (500 MHz) spectrum of compound **1** in DMSO-*d*^4^, Figure S2: ^13^C NMR (125 MHz) spectrum of compound **1** in DMSO-*d*_4_, Figure S3: ^1^H-^1^H COSY NMR (500 MHz) spectrum of compound **1** in DMSO-*d*_4_, Figure S4: ^1^H-^1^H NOESY NMR (500 MHz) spectrum of compound **1** in DMSO-*d*_4_, Figure S5: ^1^H-^1^H HSQC NMR (500 MHz) spectrum of compound **1** in DMSO-*d*_4_, Figure S6: ^1^H-^1^H HMBC NMR (500 MHz) spectrum of compound **1** in DMSO-*d*_4_, Figure S7: The HR-ESI-MS data of **1**, Figure S8: The UV spectrum of **1** in methanol, Figure S9: The CD spectrum of **1** in methanol, Figure S10: The CD spectrum of **7** in methanol, Figure S11: The CD spectrum of **11** in methanol.
