# Supplementary material for: Constituents of Coreopsis lanceolata Flower and Their Dipeptidyl Peptidase IV Inhibitory Effects
Source: Molecules. 2020 Sep 23;25(19):4370. doi: 10.3390/molecules25194370 (PMC7582822; doi:10.3390/molecules25194370)

Supporting Information

Constituents of *Coreopsis lanceolata* flower and their dipeptidyl peptidase IV inhibitory effects

Bo-Ram Kim[1,2], Sunil Babu Paudel[3], Joo-Won Nam[3], Chang Hyun Jin[1], Ik-Soo Lee[2,]* and Ah-Reum Han[1,]*

[1]*Advanced Radiation Technology Institute, Korea Atomic Energy Research Institute, Jeongeup-si, Jeollabuk-do 56212, Republic of Korea*
[2]*College of Pharmacy, Chonnam National University, Gwangju 61186, Republic of Korea*
[3]*College of Pharmacy, Yeungnam University, Gyeongsan-si, Gyeongsangbuk-do 38541, Republic of Korea*

CONTENS

**Figure S1.**	^{1}H NMR (500 MHz) spectrum of compound **1** in DMSO-*d*4	S2
**Figure S2.**	^{13}C NMR (125 MHz) spectrum of compound **1** in DMSO-*d*4	S3
**Figure S3.**	^{1}H-^{1}H COSY NMR (500 MHz) spectrum of compound **1** in DMSO-*d*4	S4
**Figure S4.**	^{1}H-^{1}H NOESY NMR (500 MHz) spectrum of compound **1** in DMSO-*d*4	S5
**Figure S5.**	^{1}H-^{1}H HSQC NMR (500 MHz) spectrum of compound **1** in DMSO-*d*4	S6
**Figure S6.**	^{1}H-^{1}H HMBC NMR (500 MHz) spectrum of compound **1** in DMSO-*d*4	S7
**Figure S7.**	The HR-ESI-MS data of **1**	S8
**Figure S8.**	The UV spectrum of **1** in methanol	S10
**Figure S9.**	The CD spectrum of **1** in methanol	S11
**Figure S10.**	The CD spectrum of **7** in methanol	S12
**Figure S11.**	The CD spectrum of **11** in methanol	S13

Figure S1. ^{1}H NMR (500 MHz) spectrum of compound **1** in DMSO-d_4.

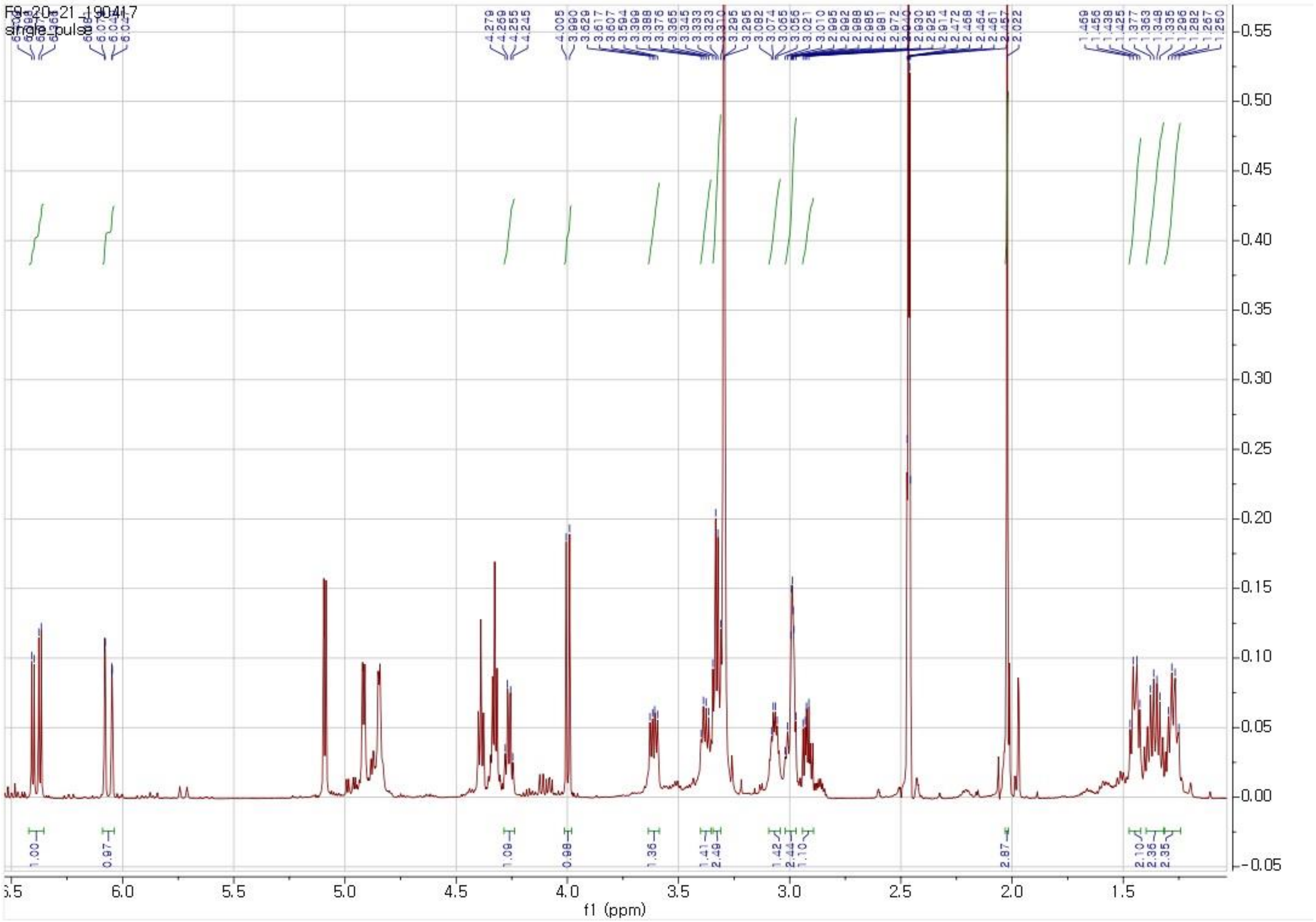

Figure S2. ^{13}C NMR (125 MHz) spectrum of compound **1** in DMSO-d_4

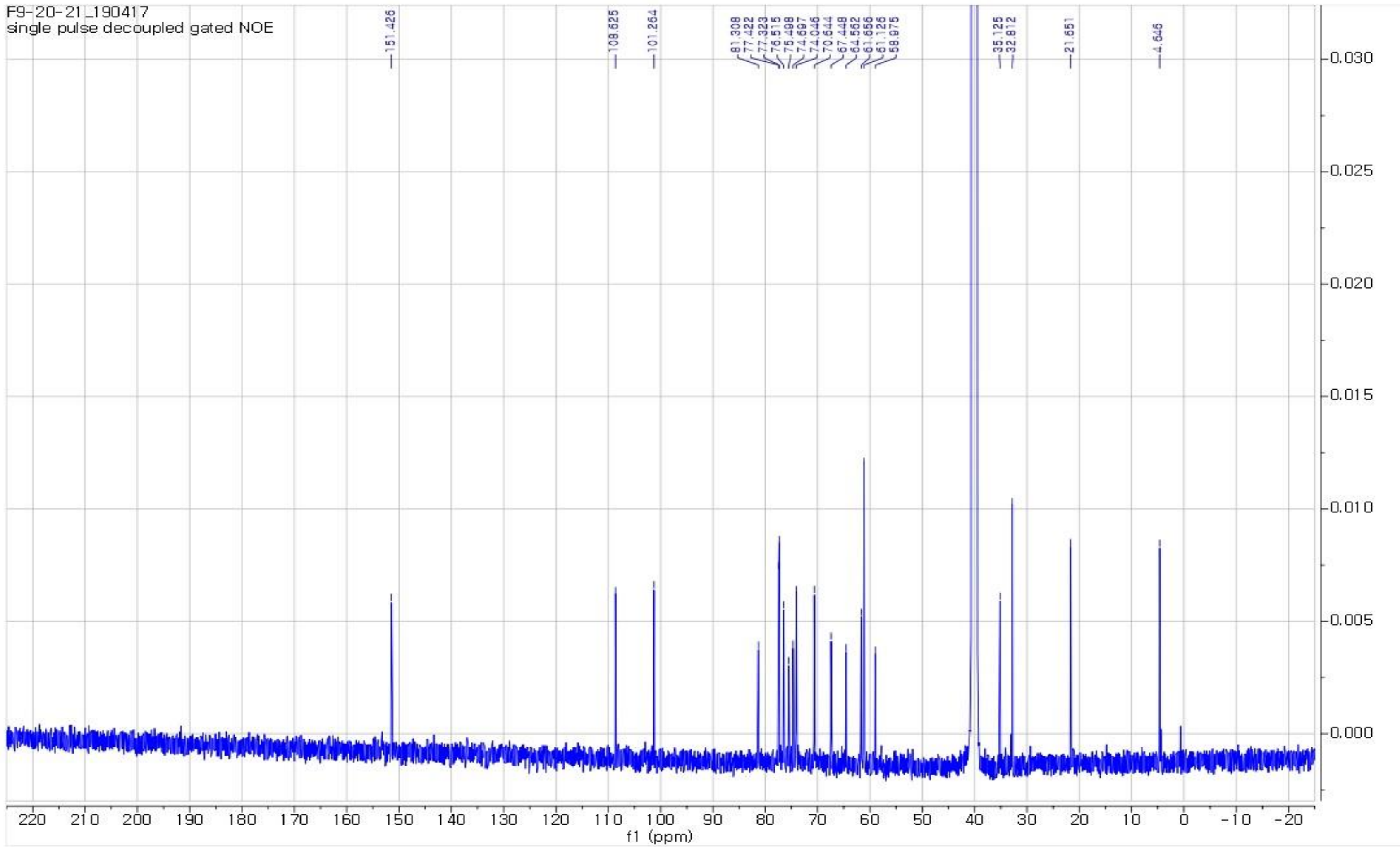

Figure S3. ^{1}H-^{1}H COSY NMR (500 MHz) spectrum of compound **1** in DMSO-d_4.

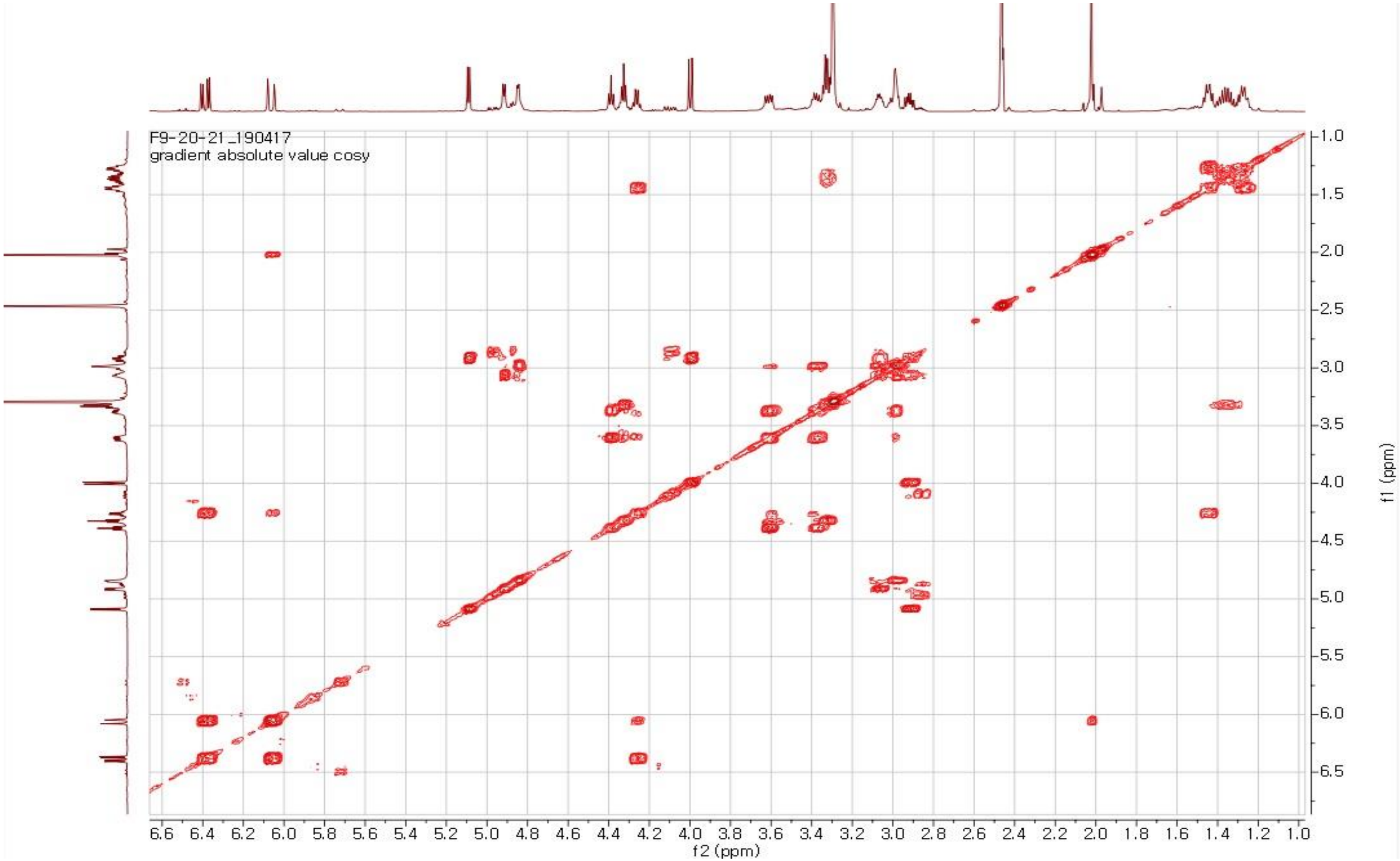

Figure S4. ^{1}H-^{1}H NOESY NMR (500 MHz) spectrum of compound **1** in DMSO-d_4.

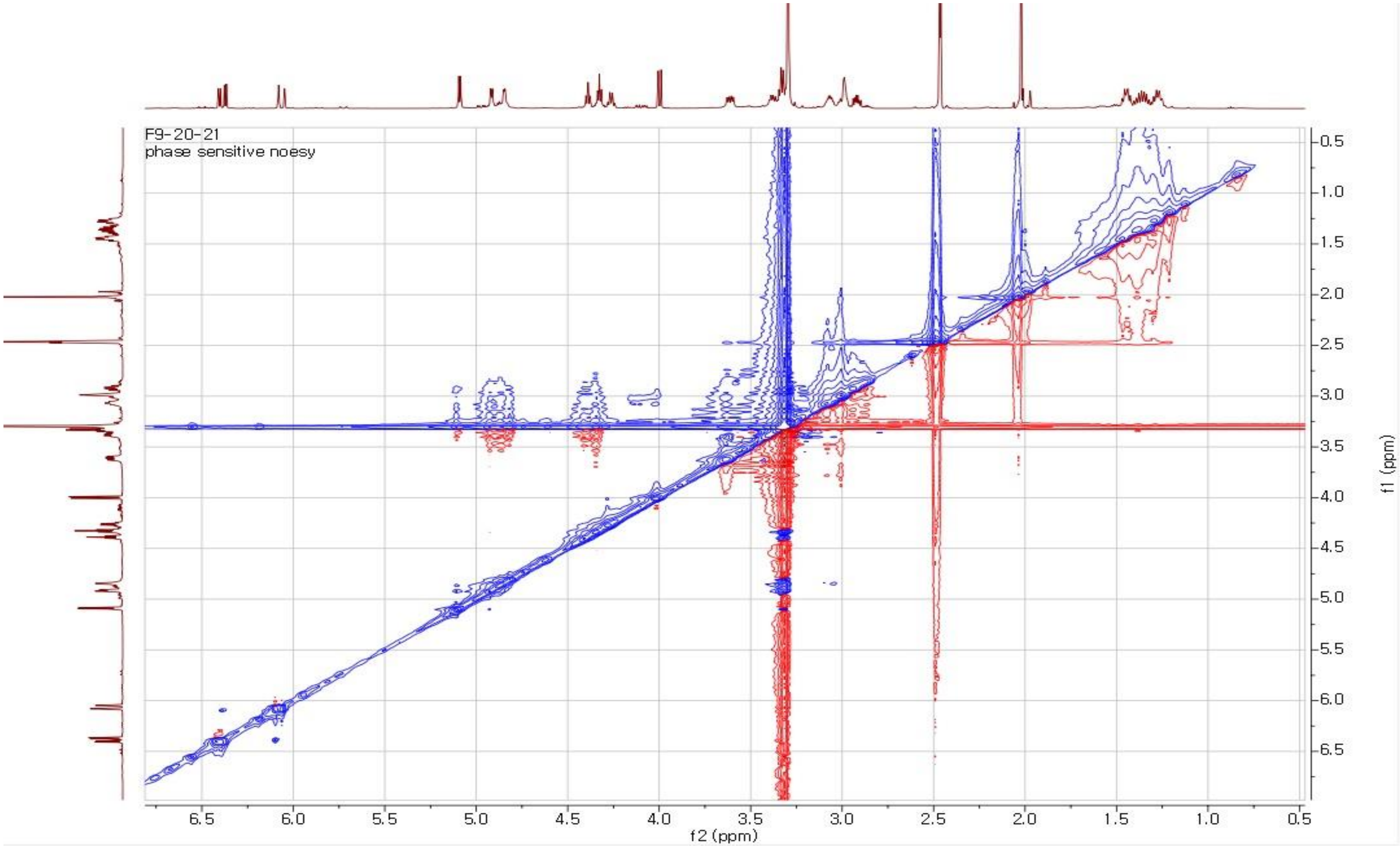

Figure S5. ^{1}H-^{1}H HSQC NMR (500 MHz) spectrum of compound **1** in DMSO-d_4.

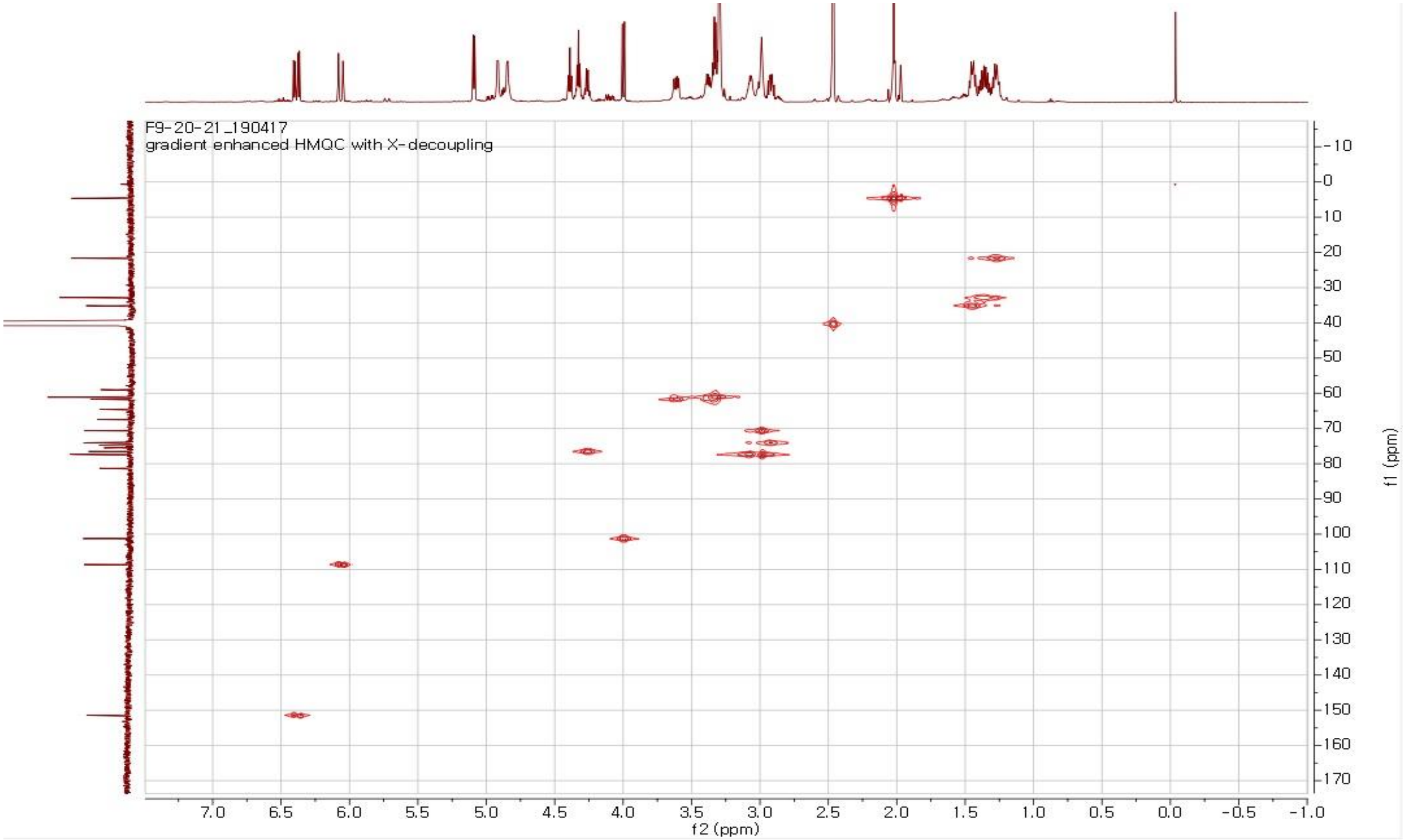

Figure S6. ^1H-^1H HMBC NMR (500 MHz) spectrum of compound **1** in DMSO-d_4.

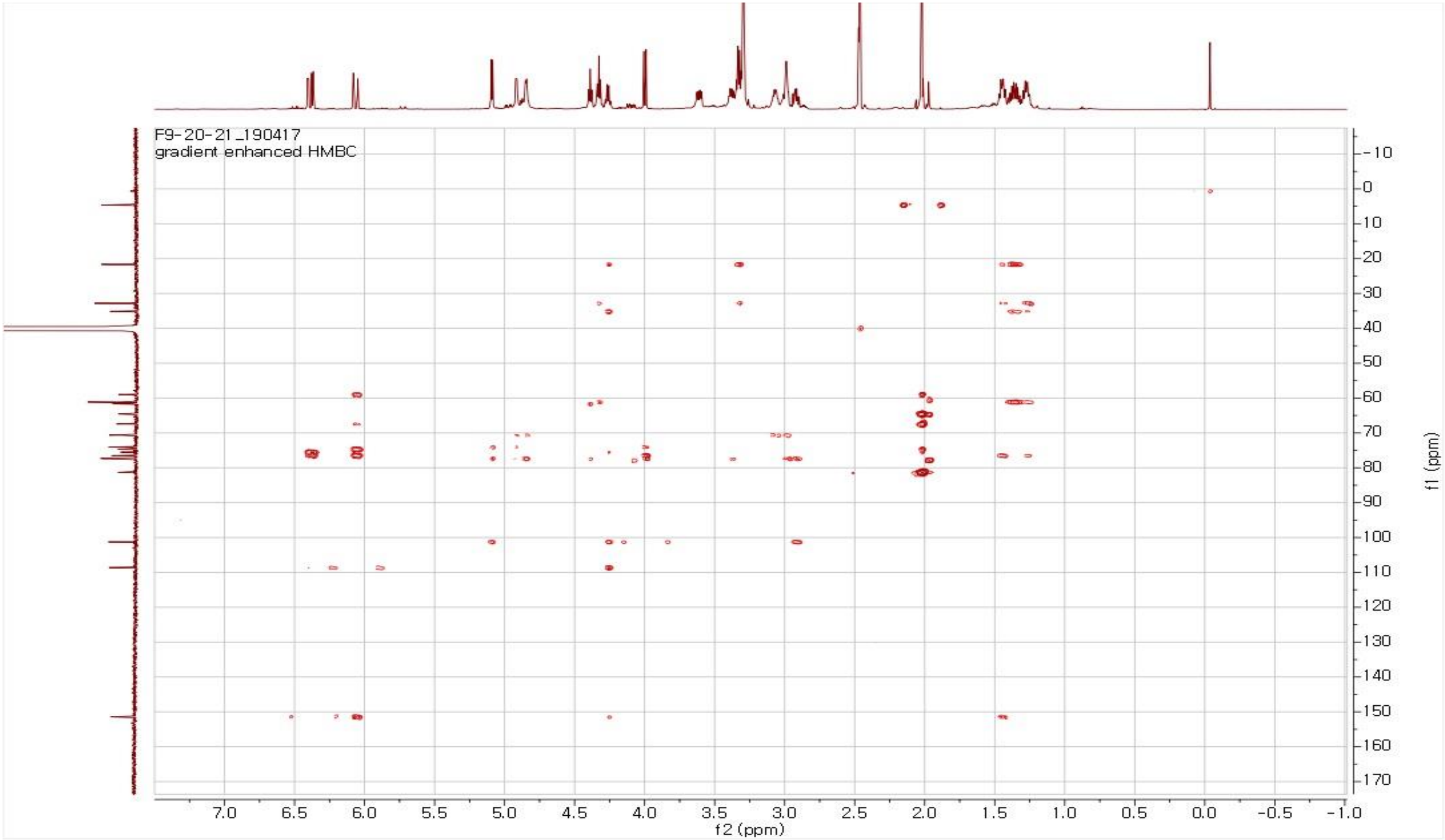

Figure S7. The HR-ESI-MS data of **1**.

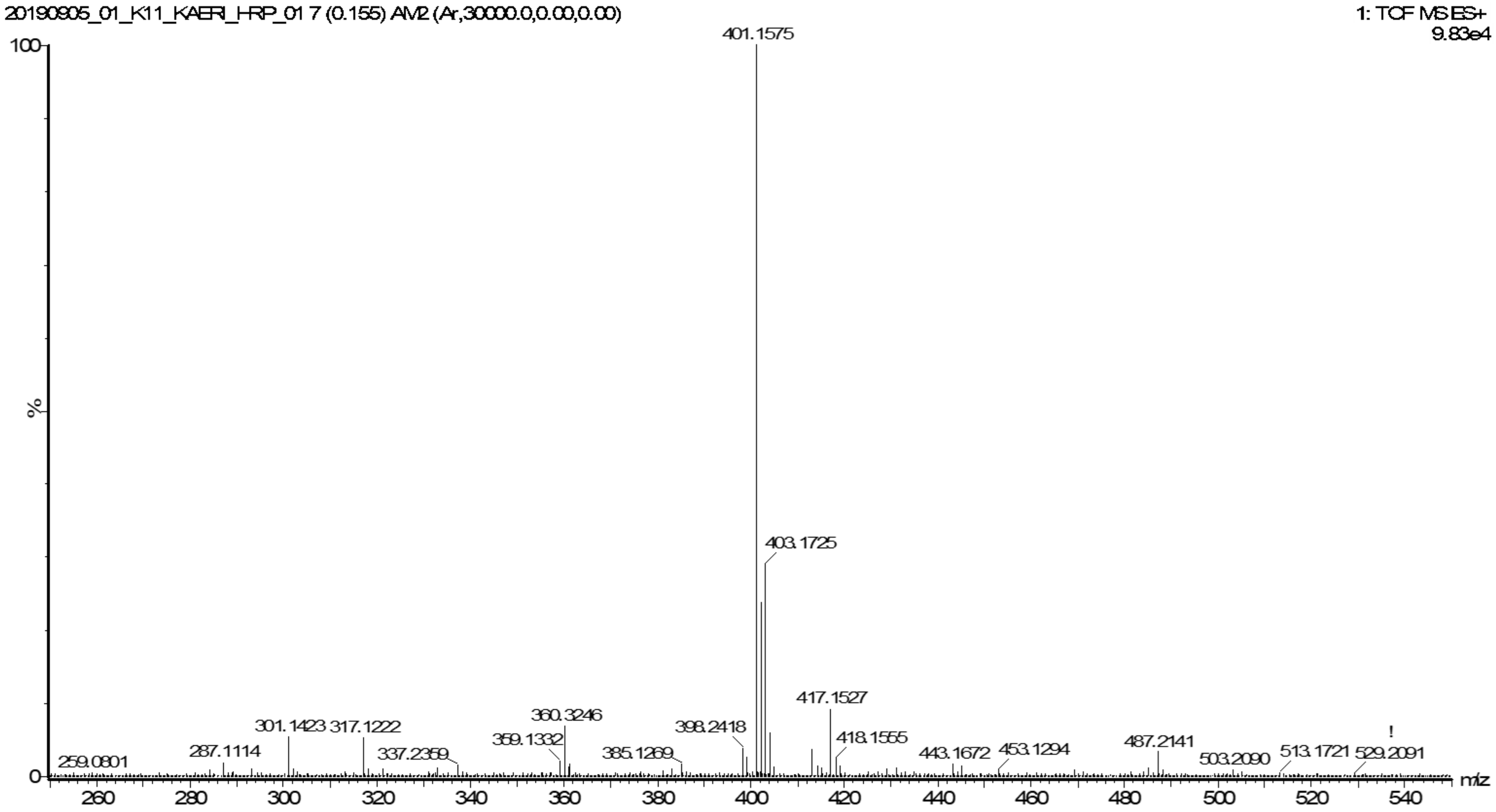

Elemental Composition Report

Single Mass Analysis
Tolerance = 5.0 PPM / DBE: min = -1.5, max = 100.0
Element prediction: Off
Number of isotope peaks used for i-FIT = 3

Monoisotopic Mass, Even Electron Ions
142 formula(e) evaluated with 1 results within limits (up to 50 closest results for each mass)
Elements Used:
C: 0-50 H: 0-50 O: 0-20 Na: 0-1

Minimum:				-1.5				
Maximum:		100.0	5.0	100.0				
Mass	Calc. Mass	mDa	PPM	DBE	i-FIT	Norm	Conf(%)	Formula
401.1575	401.1576	-0.1	-0.2	7.5	721.7	n/a	n/a	C20 H26 O7 Na

Figure S8. The UV spectrum of **1** in methanol.

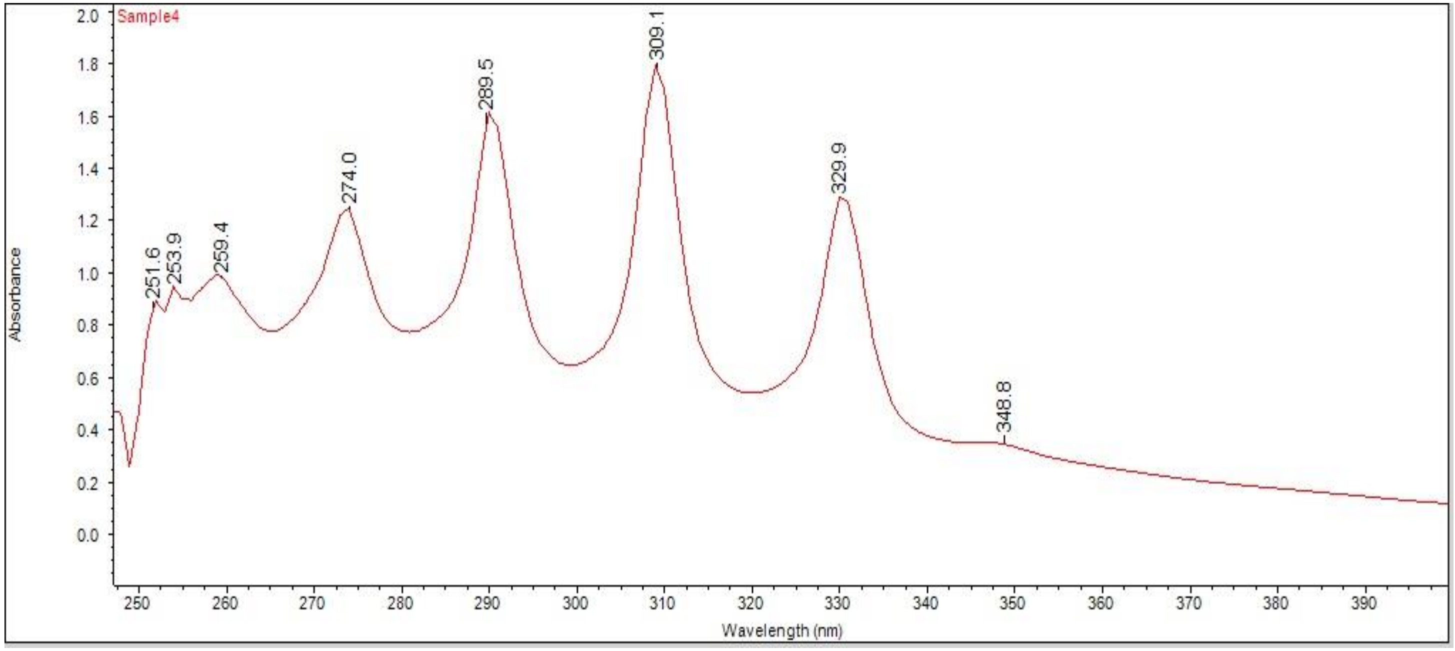

Figure S9. The CD spectrum of **1** in methanol.

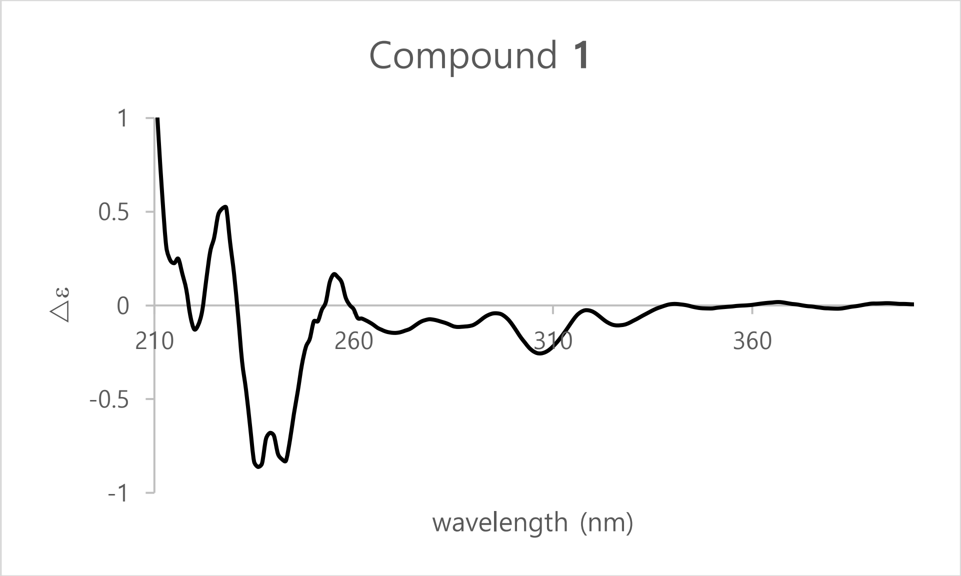

Figure S10. The CD spectrum of **7** in methanol.

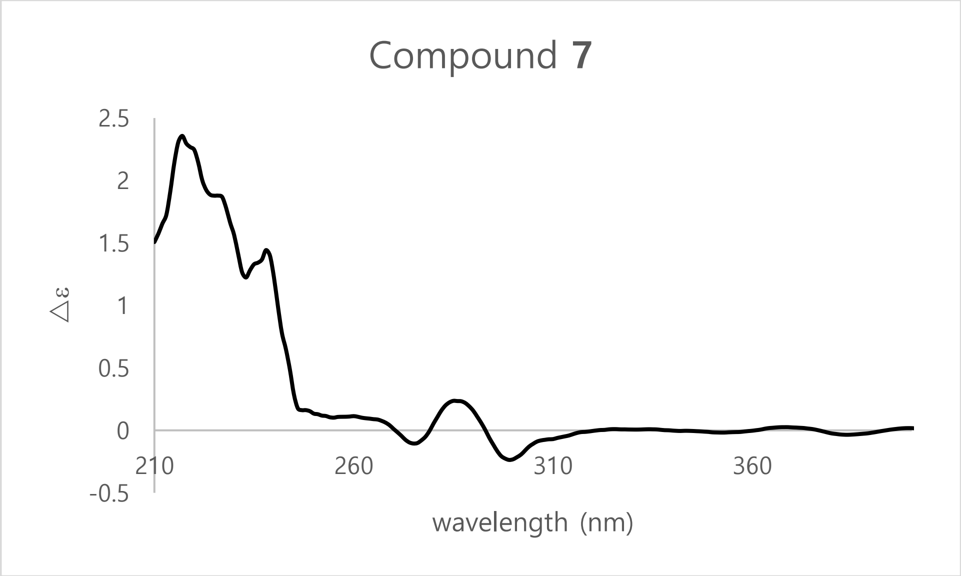

Figure S11. The CD spectrum of **11** in methanol.

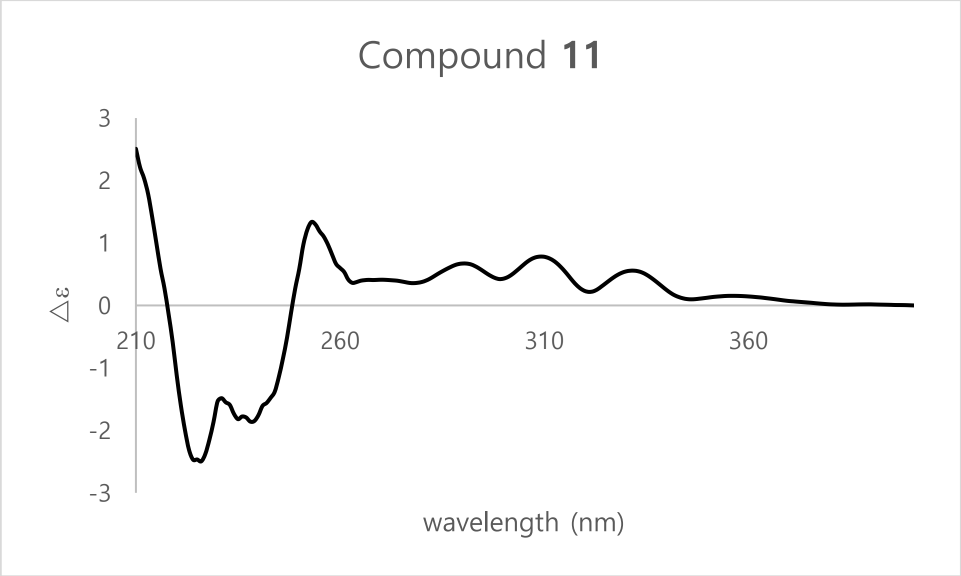

Supplement: Supplementary file 1 [file molecules-25-04370-s001.pdf]
